# Sex differences in allometry for phenotypic traits in mice indicate that females are not scaled males

**DOI:** 10.1038/s41467-022-35266-6

**Published:** 2022-12-12

**Authors:** Laura A. B. Wilson, Susanne R. K. Zajitschek, Malgorzata Lagisz, Jeremy Mason, Hamed Haselimashhadi, Shinichi Nakagawa

**Affiliations:** 1 Evolution & Ecology Research Centre, UNSW Data Science Hub, and School of Biological, Earth and Environmental Sciences, University of New South Wales, Sydney, NSW 2052 Australia; 2 School of Archaeology and Anthropology, The Australian National University, Canberra, ACT 2600 Australia; 3 School of Biological and Environmental Sciences, Liverpool John Moores University, Byrom Street, Liverpool, L3 3AF UK; 4 Melio Healthcare Ltd., City Tower, 40 Basinghall Street, London, EC2V 5DE UK; 5 European Molecular Biology Laboratory, European Bioinformatics Institute (EMBL-EBI), Wellcome Genome Campus, Hinxton, Cambridgeshire CB10 1SD UK

**Keywords:** Evolution, Zoology, Sexual dimorphism, Genetic databases, Mouse

## Abstract

Sex differences in the lifetime risk and expression of disease are well-known. Preclinical research targeted at improving treatment, increasing health span, and reducing the financial burden of health care, has mostly been conducted on male animals and cells. The extent to which sex differences in phenotypic traits are explained by sex differences in body weight remains unclear. We quantify sex differences in the allometric relationship between trait value and body weight for 363 phenotypic traits in male and female mice, recorded in >2 million measurements from the International Mouse Phenotyping Consortium. We find sex differences in allometric parameters (slope, intercept, residual SD) are common (73% traits). Body weight differences do not explain all sex differences in trait values but scaling by weight may be useful for some traits. Our results show sex differences in phenotypic traits are trait-specific, promoting case-specific approaches to drug dosage scaled by body weight in mice.

## Abstract (web summary)

Research aimed at improving healthcare has largely focused on male animals and cells. Here, the authors use data from the International Mouse Phenotyping Consortium to show that body weight does not account for all phenotypic differences between male and female mice, supporting more female-focused research.

## Introduction

A historic use of male animals in preclinical research and male participants in clinical trials has resulted in a significant bias in healthcare systems around the world^1^. The knowledge available on many diseases, their manifestation, time course and the efficacy of treatment options, is highly skewed in favour of males. The need to reach parity of the sexes in biomedical research and to conduct sex-specific analysis of research results has been widely acknowledged^2–6^. Efforts to address this issue resulted in legislative changes around clinical research, requiring female participants in government-funded clinical trials (e.g.,^7–9^). Modest improvement to rebalancing representation of the sexes in clinical trials^10–12^ has been bolstered by recent revisions to government guidelines in the US for preclinical research, requiring biological sex to be included as a study variable^13^.

Preclinical research on mice, one of the most common animal models for investigating human disease^14,15^, is fundamental for informing clinical research. These data illuminate clinically relevant pharmacological processes and enable the testing of treatment effects that would raise ethical and safety issues in humans^15^. With the growing recognition of the importance of sex in biomedicine, a sharper focus on the topic in data from mice has revealed that some of the initial assumptions and concerns surrounding use of female animals in preclinical research, such as their propensity for greater variation associated with the oestrous cycle, lack empirical support^2,16–18^.

Building on empirical studies that have sought to establish the nature of sex differences in biomedicine and to clarify the assumptions surrounding preclinical research data collected on males and generalised to females^2,18–20^, we here use an allometric framework and large phenotype data set to tackle the unresolved issue of whether sex differences in phenotypic traits in mice may be explained by sex differences in body weight. The extent to which body weight may eliminate the statistical significance of sex as an independent variable remains unclear^21^, yet is material to debate surrounding how reductions in health disparities may be effectively targeted. Data on allometric scaling relate to one of the most salient aspects of sex differences, those concerning adverse drug reactions (ADRs). Compared to men, women experience ADRs almost twice as often^22^, with the same therapeutic dose often being prescribed to both sexes^23^. Defining the allometric relationship between phenotypic trait and body weight for males and females is required to better understand whether this relationship is upheld across diverse traits and whether most observed differences are due to scaling. This will help inform whether the majority of sex-specific ADRs might be resolved by implementing weight-adjusted doses and, if not, whether the use of weight-adjusted dosing may be helpful in some circumstances at reducing sex-specific ADRs.

Studies have established that the nature of disease experience and benefits of treatment differ between men and women^24–29^, and that for many systems the observed sex differences in traits relate to differences in underlying processes rather than sex differences in body size. These differences manifest in major pillars of healthcare, impacting cost associated with care and its quality^30^. For example, the broadly divergent behaviour of male (anti-inflammatory) and female (pro-inflammatory) immune systems translates to antibody response variability, with some vaccines resulting in a stronger immune response in men compared to women^31–34^. Further, sex differences in metabolism underlie more pronounced expression of metabolic syndrome disease (e.g., obesity) phenotypes in mice and rat models^35^. These have been linked to fundamental regulatory differences in metabolic homoeostasis, impacting energy partitioning and storage. Pathophysiological differences between the sexes have also been recognised in cardiology, such that diagnostic data extracted from coronary angiograms are interpreted in a sex-specific manner^36^. In the context of drug treatment, sex differences in pharmacokinetics, related to absorption rates (e.g., gastric enzymes^37^), distribution mechanisms (e.g., plasma binding capacity^38^), and metabolism (e.g., renal elimination capacity) result in men having lower free drug concentration and higher drug clearance compared to women^38^. Likewise, sex differences in major aspects of renal function (e.g., glomerular filtration rate^39^) mean that the effects of a drug on the body are also different for men and women, which, together with pharmacokinetic parameters, translates to differences in drug efficacy and toxicity^22^. Extending across and beyond many of these systems in which sex differences have been identified, we here provide empirical data on static allometry across phenotypic traits that represent preclinical parameters (e.g., immunology, metabolism, morphology) in a disease model animal (mouse). We aim to clarify if, and the extent to which, trait values for male mice may be scaled to match those of female mice.

Here, we show that sex differences in body weight do not explain all differences in phenotypic traits between male and female mice, highlighting that sex differences in allometry may impact study outcomes in biomedicine. We adopt the framework of static allometry, the measurement of trait covariation among individuals of different size at the same developmental stage, following Huxley^40,41^ who proposed an equation to model simple allometry. This equation expresses the growth of two traits, *x* and *y*, when regulated by a common growth parameter: *y* = *ax*^*b*^ or equivalently, log *y* = log(*a*) + *b* log(*y*), where the ratios between the components of the growth rates of *y* and *x* correspond to intercept log(*a*) and a slope *b*^42^. We quantify the relationship between phenotypic trait and body weight in males and females, statistically evaluating scenarios that describe the magnitude and patterning of sex differences across 363 traits in over 2 million mice from the International Mouse Phenotyping Consortium^43^ (IMPC, www.mousephenotype.org). We discuss these data considering the discourse on the generalisation of male data in preclinical research^44^, as well as their evolutionary implications, leveraging a large, wildtype data set to illuminate trends in static allometry. Consideration of the evolutionary context surrounding sex differences may augment understanding of how disease state phenotypes emerge or persist in a population^45,46^.

## Results

### Data characteristics

Following initial data cleaning and filtering procedures, the data set comprised 363 phenotypic traits with a mean sample size of 2866 mice per trait (*n* = 2,080,767). Representation of males and females was highly similar across most phenotypic traits, with fewer than 15% of traits (53/363) displaying greater than 5% difference in sample size between the sexes. The traits were collated into nine functional groupings following Zajitschek et al.^18^ (see “Methods”): behaviour (85 traits, *n* = 440,491), eye (40 traits, *n* = 21,871), hearing (21 traits, *n* = 273,715), heart (31 traits, *n* = 233,772), haematology (24 traits, *n* = 291,214), immunology (99 traits, *n* = 92,130), metabolism (8 traits, *n* = 108,788), morphology (21 traits, *n* = 287,420), and physiology (34 traits, *n* = 331,366).

The 363 phenotypic traits were further filtered for non-independence of traits, so that *p* values were merged for traits that were related to one another, resulting in a reduced data set of 219 traits, with a mean sample size of 3530 males and 3598 females per trait.

### Linear mixed-effects models for static allometry

**Fig. 1:**
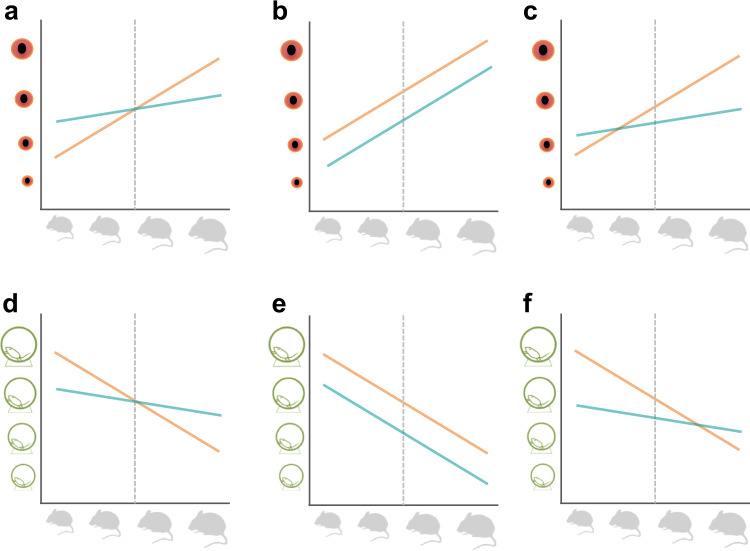
Examples of scenarios of sex differences in the allometric relationship between phenotypic trait and body weight. Top row shows a hypothetical positive relationship between body weight (x-axis) and eye size (y-axis) and the bottom row shows a negative relationship between body size and activity level (y-axis). Body weights are scaled and centred so that the intercept is at the trait mean represented by a grey dashed line. A series of scenarios are illustrated as follows. **a** The sexes show different positive slopes but the same intercept. **b** Both sexes have the same positive slope but different intercepts. **c** The sexes show different positive slopes and different intercepts. **d** The sexes show different negative slopes but the same intercept. **e** Both sexes have the same negative slope but different intercepts. **f** The sexes have different negative slopes and different intercepts.

Our linear mixed-effects models, which included sub-strain as a random effect, indicated that 11 out of 219 traits (5%) (17/363 traits for unmerged *p* values) are associated with scenario A (different slope, same intercept, Fig. 1a, d); most of these traits belonged to immunology and heart functional groups. Scenario B (same slope, different intercept, Fig. 1b, e) was supported for 93/219 (42%) traits (154/363 traits for unmerged *p* values). For scenario C (different slope, different intercept, Fig. 1c, f), 57/219 (26%) traits were categorised as consistent (70/363 traits for unmerged *p* values), and the remaining 58/219 (26%) traits showed no significant differences in slope and intercept between males and females. Overall, when a statistically significant difference in allometric pattern was present between the sexes, intercept differences appeared more common than slope differences (42% compared to 5% traits), however both slope and intercept differences were also common (26%). Just over a quarter of traits showed no significant differences between males and females, indicating that, for most traits, sex differences in allometric patterning represent a significant source of variation in trait values. All slopes presented as hypo-allometric with slope values of <1.

Our comparisons of model fit for our applied model, including sub-strain of mice as a random factor, and a model without sub-strain, indicated that accounting for sub-strain variation resulted in a significantly greater fit of our model for 155 out of 248 traits (63%) in the unmerged comparison. The functional groups with the greatest number of traits with significant delta AIC values (δAIC), indicating improved model fit when sub-strain was included as a random factor, were heart (25/31 traits, 81%) and immunology (51/63 traits, 81%). Model fit was significantly greater for 98 out of 154 traits (64%) in the merged comparison.

Taken together, traits in all functional groups showed statistically significant (*α* = 0.05) sex differences. Slope differences between the sexes (scenario A) and intercept differences between the sexes (Scenario B) are most common in behaviour, immunology and physiology groups. Traits exhibiting both slope and intercept differences between the sexes (scenario C) were most commonly found in the behaviour, physiology and haematology functional groups. Non-significant differences in slope and intercept were most common among traits in the behaviour and eye functional groups.

### Sex bias in allometric parameters

**Fig. 2:**
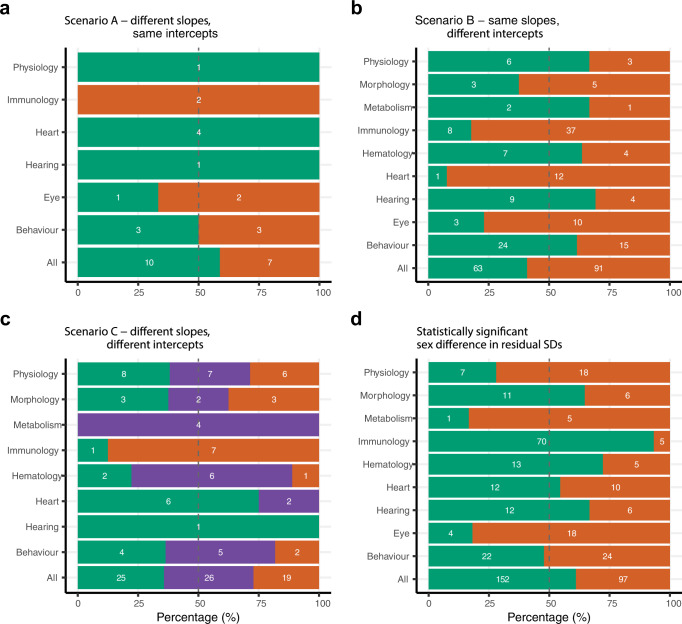
Sex biases for phenotypic traits in mice, arranged in functional groups. Sex bias represents a greater parameter value (slope, intercept, variance) in one sex compared to the other. Colours represent significant differences in trait values between the sexes (green = male biased, orange = female biased). The number of traits that are either female biased (relative length of orange bars) or male biased (relative length of green bars) are expressed as a percentage of the total number of traits in the corresponding group. Numbers inside the green bars represent the numbers of traits that show male bias within a given group of traits, values inside the orange bars represent the number of female biased traits, and those inside the purple bars represent a combination of female bias (for intercept or slope) and male bias (for intercept or slope). **a** Differences between the sexes for slope. **b** Differences between the sexes for intercept. **c** Differences between the sexes for slope and intercept, including traits with mixed (purple) significant differences (i.e., male-biased significant slope and female-biased significant intercept, or female-biased significant slope and male-biased significant intercept). **d** Bias in statistically significant difference in variance (residual SD) between the sexes.

Values for sex bias represent the number of traits that showed greater parameter value when male and female mice differed significantly. That is, we counted which sex displayed the greater intercept, slope and higher magnitude of variance. Sex bias in the slope and intercept values, in addition to the magnitude of variance (residual SD), showed considerable variability across functional groups, suggesting trait-specific patterning of sex differences. For scenario A, representing traits with significant differences in slope, most traits showed greater slope magnitudes for males (*n* = 10 traits), rather than for females (*n* = 7 traits) (Fig. 2a). For scenario B, females showed greater intercept magnitudes for heart, morphology, immunology, and eye functional groups (*n* = 91 traits), whereas males showed greater intercepts for traits in physiology, metabolism, haematology, behaviour and hearing functional groups (*n* = 63 traits) (Fig. 2b). Overall sex bias (63 male traits: 91 female traits, Fig. 2b) was slightly greater for intercept differences, compared to slope differences (10 male traits: 6 female traits, Fig. 2a). Scenario C, which represents significant slope and intercept parameter differences between the sexes, was predominated by mixed bias across three out of nine functional groups (*n* = 26 traits), indicating that traits most frequently showed a mixture of directional differences in bias, comprising a combination of male bias in one parameter (slope or intercept) and female bias in the other parameter (slope or intercept) (Fig. 2c). Immunology and hearing-related traits represent an exception under scenario C, whereby traits with significant differences between the sexes did not show a mixed bias for slope and intercept values, consistent with few sex differences among traits. Across functional groups, male bias is slightly more common (5 groups) than female bias (4 groups) for statistically significant sex difference in residual SD, indicating that where traits show differences between the sexes, it is more common for males to be more variable than females, than vice versa (Fig. 2d) (152 male traits: 97 female traits).

### Meta-analysis and meta-regression of sex differences in slope, intercept and variance

**Fig. 3:**
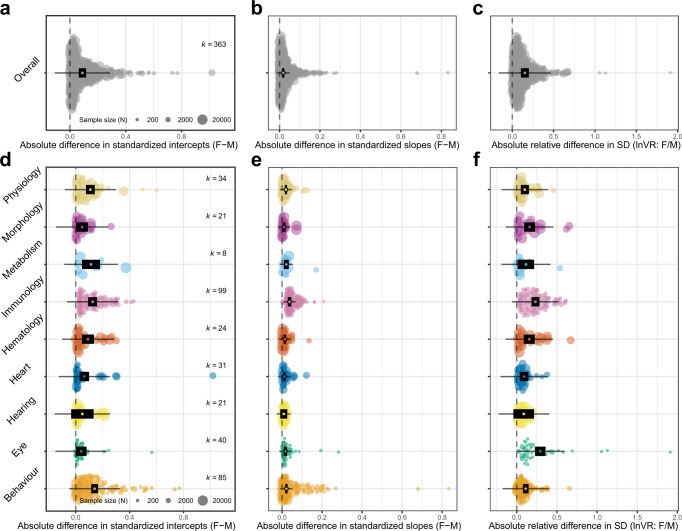
Orchard plots illustrating results of multivariate meta-analysis. Orchard plots show model point estimate (black open ellipse) and associated confidence interval (CIs) (thick black horizontal line), 95% prediction intervals (PIs) (thin black horizontal line; PI represents heterogeneity), and individual effect sizes (filled circle), which are scaled by their sample size (N), the number of mice included per trait. The number of effect sizes (number of phenotypic traits) is represented by *k*. **a** Overall difference between male and female absolute values for allometric intercept. **b** Overall difference between male and female absolute values for allometric slope. **c** Overall difference between male and female values for residual variance (SD). **d** Difference between male and female values for allometric intercept, separated by functional group. **e** Difference between male and female values for allometric slope, separated by functional group. **f** Difference between male and female values for residual variance (SD), separated by functional group.

Multi-level meta-analysis of absolute values in allometric slope and intercept, and variance revealed significant differences between the sexes (Fig. 3a–c). For overall comparisons (Fig. 3a–c), the effect sizes ranged from significant (i.e., confidence interval [CI] not overlapping with zero) point estimates of 0.089 [0.063–0.115, CI] (Fig. 3a) for sex differences in intercept to 0.152 [0.1.5–0.200, CI] (Supplementary Table 1) for differences in residual SD (Fig. 3c). Across functional groups, there was variability in the magnitude of absolute difference between the sexes, both within parameters (i.e., intercept) and across parameters. For absolute differences in intercept, traits within the behaviour functional group showed the greatest model point estimate difference between males and females (0.140 [0.115–0.166, CI] (Supplementary Table 1). Effect sizes were significant for all groups (Supplementary Table 1), except traits within the hearing group, which showed the smallest magnitude of difference (Fig. 3d), with a point estimate of 0.049 [−0.034 to 0.132, CI] (Supplementary Table 1). For differences in slope, except for the hearing group, all categories showed significant effect sizes (Supplementary Table 1). The largest model point estimate difference was observed for immunology traits (0.037 [0.029–0.046, CI]), being almost four times greater than the effect size for morphology traits (0.010 [0.002–0.017, CI]) (Fig. 3e, Supplementary Table 1).

For the relative difference in residual SD, the greatest effect sizes were found for eye (0.292 [0.228–0.357, CI]) and immunology traits (0.234 [0.184–0.283, CI]), between two and three times greater than traits within the heart group, which showed the lowest significant effect size (0.091 [0.038–0.145]) (Supplementary Table 1). Hearing traits were most similar in SD values between the sexes, having a non-significant effect size (0.087 [−0.04 to 0.216, CI]) (Fig. 3f, Supplementary Table 1). Overall, across all parameters (intercept, slope, SD and model fit), confidence intervals (CIs) for hearing traits were the only ones to consistently overlap with zero, showing no statistically significant difference between the sexes (Fig. 3d–f, Supplementary Table 1). For traits within a given functional group, there was considerable variability in the magnitude of difference between the sexes. For sex differences in intercept, inter-trait variability was highest within physiology, metabolism and behaviour groups (Fig. 3d), whereas slope differences showed most inter-trait variability for eye and behaviour traits (Fig. 3e). Relative difference in SD was most variable among traits in the eye group (Fig. 3f).

### Relationship between slope/intercept and residual variance

**Fig. 4:**
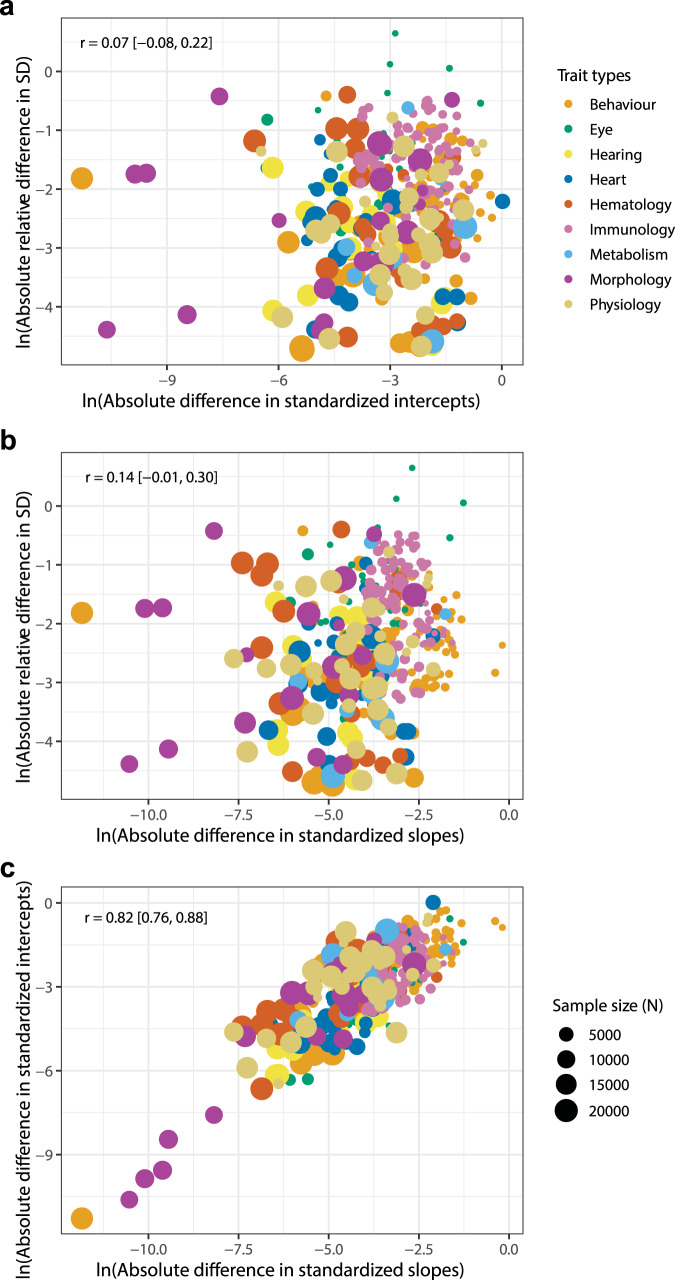
Bivariate ordinations of log absolute difference between males and females for allometric variables. Plots show biological traits collated into nine functional groups (i.e., trait types, represented as different circle colours). Individual effect sizes (circles) are scaled by their sample size (N), the number of mice included per trait. **a** Relationship between allometric intercept and residual SD. **b** Relationship between allometric slope and residual SD. **c** Relationship between allometric slope and intercept.

Our quad-variate meta-regressions and ordinations of the relationships between slope, intercept and residual variance (Fig. 4) revealed weak, non-significant, correlations between either slope or intercept and residual variance (*r* = 0.07–0.14, Fig. 4a, b), indicating that a greater magnitude of difference between the sexes in either slope or intercept parameter is not strongly associated with greater trait variance. In contrast, absolute differences between the sexes in slope and intercept are strongly and significantly correlated (*r* = 0.82, Fig. 4c), indicating that in cases where there are significant differences in trait values for males and females, should a difference in intercept be present, this is likely accompanied by a difference in allometric slope.

## Discussion

Most current medical guidelines are not sex-specific, being informed by preclinical studies that have been conducted only on male animals^4,10,47^ under the assumption that the results are equally applicable to females, or that the female phenotype represents a smaller body size version of the male phenotype^48,49^. We find that 73% of traits are dimorphic, either in slope, intercept or both slope and intercept, and that among these 42% of traits show scaling differences between male and female mice. Scaling differences are not supported across all phenotypic traits, indicating that body weight alone is not enough to explain sex differences in phenotypic traits but that it may be useful for some traits.

In an era where personalised medicine interventions are within reach and patient-specific solutions represent a realisable frontier in healthcare (e.g.,^50–52^) it is now well recognised that sex-based data are much needed to advance care in an equitable and effective manner. As studies that illuminate the presence and importance of sex differences continue to emerge, many experimental set-ups that use both sexes continue to eschew downstream testing for sex differences, in part due to perceived inflation of sample size required for such analyses^48,53–55^.

Explicit male-female comparisons are needed to clarify the nature of sex differences^47,56^. Here we address this issue through a novel meta-analytical focus on identifying and characterising allometric scaling relationships for biological traits on a broad scale. Our meta-analytical results recover significant effect sizes for sex differences in slope, intercept and residual SD across all functional groups, with the only exception being for traits assigned to the hearing group, which are similar (CI overlapping zero) for males and females across all allometric parameters analysed (Supplementary Table 1, Supplementary Data 1, Supplementary Data 2). Standardised mean differences (SMD, or Cohen’s *d*) indicated a medium effect size of sex difference for 9% of traits (33/363), mostly within behaviour, eye and physiology groups, and a large effect size for 7% of traits (25/363), mostly among behaviour, metabolism, morphology and physiology groups (Supplementary Data 2). An additional 29% (106/363) of traits, distributed across all functional groups, showed a small effect size (SMD > 0.2), and the remaining traits had an SMD value of below 0.2 in magnitude (Supplementary Data 2). We identify slope parameter (*b*) differences between the sexes and find these are mostly associated with significant differences in intercept value (Fig. 4c). Where a significant difference between male and female slope is present, 38% of slopes are steeper in females and 62% of slopes are steeper in males (Supplementary Data 1). Where females have steeper slopes, ignoring the difference in slopes between the sexes would result in missing between 0.3–32.6% of trait variance (average = 8.9%) in those traits, mostly belonging to physiology and behaviour and eye groups (Supplementary Data 1). Similarly, where males have steeper slopes, ignoring the difference in slopes between the sexes would fail to capture 0.3–46.1% of variance in those traits, most markedly for morphology, haematology and heart groups (Supplementary Data 1).

We demonstrate that the relationship between trait and body mass in mice differs fundamentally in mode (i.e., change in inter-trait covariance) between the sexes and that dimorphism cannot be fully explained by a magnitude shift in intercept value, as would be predicted should female phenotype represent a scaled version of male phenotype. For traits where there are significant differences in both slope and intercept between the sexes, it is common for a mixed scenario (male-biased significant slope and female-biased significant intercept, or female-biased significant slope and male-biased significant intercept) to occur (26%), meaning a female value cannot be predicted based on an allometric coefficient extracted from regression data collected on males. Further, we find a male bias in residual SD, indicating greater variance in males, for traits in morphology, immunology, haematology, heart, and hearing functional groups (5 out of 9 functional groups).

Our findings of trait-specific allometric patterns complement recent evidence that supports a complex, trait-specific patterning of sex differences in markers routinely recorded in animal research^18,20,57^. Previous studies using phenotypic traits from the International Mouse Phenotyping Consortium have identified that sexual dimorphism is prevalent among phenotyping parameters^20^, and moreover that, contrary to long-held assumption, neither females nor males show greater trait variability.

We asked the question of whether all or some sex differences in phenotypic traits are due to differences in body weight, which has implications for drug therapy, and specifically data surrounding the efficacy of drug dosing scaled by body weight.

There exist known sex differences in drug prescription prevalence and usage patterns, as well as response to drug therapy^58,59^. The same therapeutic regimen can elicit different responses due to sex-specific variance in pharmacokinetics and pharmacodynamics profiles (e.g.,^60,61^), arising from underlying physiologic differences. These include, for example, significantly dimorphic traits captured among the physiology group in our analysis, such as iron^62^ and body temperature^63^, among the morphology group, such as lean mass and fat mass^58^, and among the heart functional group, such as QT interval (time between Q wave and T wave)^64^. Further, women are 50–75% more likely to experience Adverse Drug Reactions (ADRs)^65^, although these are not fully explained^23^. Women may be at increased risk of ADRs because they are prescribed more drugs than men, however women are usually prescribed drugs at the same dose as men, meaning that they receive a higher dose relative to body weight in most cases. Scaling of doses on a milligram/kilogram body weight basis has been recommended as a pathway to reducing ADRs^22^, particularly for drugs that exhibit a steep dose–response curve^66^. Indeed, sex differences in ADRs have been argued to be the result of body weight rather than sex, per se^21^. For both assertions to be supported, we would expect to observe a scenario (here, scenario B) whereby most or all phenotypic traits exhibit a scaled relationship between males and females, as a function of body weight.

Our results indicate that 42% traits follow scenario B, with many traits (26%) also supporting a sex- and trait-specific relationship between weight and phenotypic trait. This aligns with evidence that weight-corrected pharmacokinetics are not directly comparable in men and women^22,67^, and that many sex differences in ADRs persist after body weight correction^68,69^. Nevertheless, the Food and Drug Administration (FDA) has recommended dosage changes for women (e.g., sleep drug zolpidem^70^) and weight-adjusted dosing of some drugs, such as antifungal drugs and antihypertensive drugs, which appear to ameliorate sex differences in pharmacokinetics^71,72^. Our results are consistent with support for scaling in some circumstances, the greatest number of these traits occur in the immunology and heart groups, which contain parameters most closely relevant to pharmacokinetic and pharmacodynamic factors. Whilst acknowledging that there are known cases where mouse models are unable to capture the human response to therapeutic drugs (e.g., cancer treatment^73^), we suggest traits in scenario B as candidates for further investigation in weight-adjusted dosing. If weight-adjusted dosing is supported based on intercept scaling (scenario B), the extent to which variation in the allometric relationship may reduce the efficacy of such an approach also requires consideration. This is measured by model fit in our analyses, whereby greater model fit equates to more of the variance in phenotypic traits for males and females being explained by body weight. For those traits that scale (scenario B), the most useful candidates for dose-scaling are therefore likely to be captured by those traits with the highest model fit (Zr, transformed from R^2^ marginal) (Supplementary Fig. 2). These include morphological traits such as organ mass (*R*^2^ marginal = 0.82) and lean mass (R^2^ marginal = 0.71), as well as physiological and metabolic traits associated with lipid and glucose parameters (e.g., HDL cholesterol, R^2^ marginal = 0.60; area under glucose response curve, R^2^ marginal = 0.50). Traits with lower model fit may scale, but a considerable amount of variance in the phenotypic trait is not explained by body weight, therefore scaling may not achieve the desired clinical outcome. Traits within scenario B that meet this criterion are most frequently found in the eye, morphology and physiology groups.

We suggest that where there exists an association between sex and dose, dose–response curves are likely to be sex-specific and clarification of this relationship would be supported (e.g., using meta-analysis^74^). Since many drugs are withdrawn from the market due to risks of ADRs in women, meta-analytic approaches to illuminating sex-specific dose–response curves represents a viable opportunity to reducing the number of ADRs and reaching an important target set by precision medicine^75^. We further note that behavioural factors, such as differences in health-seeking behaviours and prescription patterns, may relate to a higher prevalence of use for most therapeutic drugs in women as compared to men^59,76^, and are not captured by our data on mice.

Established as a foundational descriptor of morphological variation at ontogenetic, population and evolutionary levels^77,78^, allometry may channel phenotypic variation in fixed directions, defining scaling relationships that persist across large evolutionary timescales. For example, craniofacial variation among mammals has been observed to be constrained by allometry, such that small mammals have shorter faces than do larger ones^79^. Conversely, allometry may facilitate morphological diversification, acting as a line of ‘evolutionary least resistance’, allowing for new morphotypes to originate relatively rapidly among closely related species^42,80^. These pathways (allometric constraint vs allometric facilitation) may be a start point for exploring how sex differences in disease phenotypes arise, data that have been cited as a potential unexploited resource relevant for the development of new therapies^81^. Studies of static allometry, as examined herein, have revealed low levels of intraspecific variation in allometric slope, which explains only a small proportion of variation in size^82^, compared to variation in allometric intercept^83^. Moreover, traits under sexual selection have also revealed low magnitudes of allometric slope change under artificial selection experiments^84^ and in wild populations^85^, whereas intercept changes appear clear and heritable. Features of the developmental system have previously been considered to act as an internal constraint^40,86^, whereas more recent interpretations suggest that external constraint (selection) more likely acts to maintain slope invariance at the static level^42^, which is consistent with data showing that variation occurs instead at the ontogenetic level, i.e. growth rate and ontogenetic allometric slope are evolvable (e.g.,^87–89^). Broadly consistent with other static allometric studies, we find that where differences in allometry are present, significant intercept shifts alone are more common than are significant slope shifts (Fig. 2a compared to 2b). Aside from the evolutionary implications—that allometric slope likely does not have a high evolvability, or capacity to evolve—many of the traits examined here may show a low level of sex difference in slope because the sexes are both experiencing the same selective pressure to maintain functional size relationships across different body sizes.

Our meta-analytic results build a narrative of complexity in sex-based trait interactions and promote a case-specific approach to preclinical research that seeks to inform drug discovery, development and dosage. Using a model that includes sub-strains of mice, across a diverse set of phenotypic traits we show that differences in body weight are not sufficient to explain sex differences in trait values, but scaling differences are common and body weight scaling may be helpful in some traits. Our results evidence the plasticity of static allometry, revealing a pathway for sex variation in phenotypic traits that may be generalisable beyond Rodentia and underlie the patterning of phenotypic space on a broader scale. Sex differences in allometry may likely influence study outcomes in biomedicine.

## Methods

### Data compilation and filtering

We conducted all data procedures, along with statistical analyses, in the R environment v. 4.1.3^90^ (data set and scripts available on Zenodo^91^). We compiled our data set from the International Mouse Phenotyping Consortium (IMPC) (www.mousephenotype.org, IMPC data release 10.1 June 2019), accessed in October 2019. These represent traits recorded in a high-throughput phenotyping setting whereby standard operating procedures (SOPs) are implemented in a pipeline concept. The phenotypic traits represent biomarkers used for the study of disease phenotypes (see ref. 20), collated into the following nine functional groups: behaviour, eye, hearing, heart, haematology, immunology, metabolism, morphology, and physiology, which are the IMPC’s original categorisation (also previously used in Zajitschek et al.^18^). These groupings were assigned in relation to the description of the procedure undertaken for data point collection and following the categorisation of pipeline events at adult stage, detailed in the International Mouse Phenotyping Resource of Standardised Screens (IMPReSS, https://www.mousephenotype.org/impress/index). Sex was operationalized using a standard operating procedure for all live pups, consistent across all data, and part of the primary viability screen in the pipeline. Mice were sexed based on the morphology of the external genitals.

For the initial data set, data points were collated for adult wildtype mice only, filtering to include non-categorical phenotypic trait values for which covariate information on sex and body weight were available. Note that all mice were from the C57BL/6 strain, but they come from seven sub-strains. This initial data set comprised of 2,866,345 data points for 419 traits. A series of data cleaning procedures were implemented to remove data points with missing body weight, zero values for a phenotypic trait and duplicated specimen IDs. Data filtering was conducted using the R package dplyr v.1.0.7^92^. The resulting data set comprised 2,118,370 data points for 379 phenotypic traits, all of which had corresponding body weight data, enabling us to estimate an allometric relationship between a trait of interest and body weight. Of these, 89 traits (24%) were on the interval scale, and were therefore adjusted to be on the ratio scale. For each phenotypic trait, we had the following variables (covariates): phenotyping centre name (location where experimental data were collected), external sample ID (animal ID), metadata group (identifier for experimental conditions in place during the experiment), sex (male/female), weight (body weight in grams), weight days old (day on which weight was recorded), strain name (identifier character for sub-strains of mice), procedure name (description of the experimental procedure as in IMPReSS), parameter name (description of the recorded parameter as in IMPReSS), and data point (phenotypic trait measurement—response variable).

### Linear mixed-effects model for static allometry

The static form of allometry, the covariation of a trait with size as measured across a population of adults within a single species^78^, was quantified using a linear mixed-effects model approach^93^. Within this framework, the relationship between phenotypic trait value and body weight, accounting for random effects associated with sub-strain, assignment to a metadata group and batch (defined as the date when the measurements are collected), was quantified for each of the 379 traits. We use body weight as opposed to adiposity (trait ~ fat mass) because, although there are sex differences in prevalence of obesity^94^ which may limit body weight differences between males and females, body mass is the clinical measure used in studies assessing drug dose–response and collected in healthcare settings. Models were constructed using the function *lme* in the R package nlme v. 3.1-153^95^ and applied to each phenotypic trait separately. We applied a grand-mean centring (gmc) to the continuous predictor (i.e., weight); in this way, the intercepts (*x* = 0) for each sex provide the predicted value for a female or a male of similar weight. The applied model was:

log (data point) ~ gmc(log (weight)) * sex + (1 | batch) + (gmc(log (weight)) | metadata group) + (gmc(log (weight)) | sub-strain)

The random factor ‘batch’ labelled a cohort of mice that went through a procedure on the same day (see ref. 20), ‘metadata group’ represented occasions when procedural parameters were changed (e.g., different instruments, different observers and different settings) and strain represents the strain identifier (e.g., C57BL/6N) for each mouse. These three random factors along with the ‘weight’ random slopes would reduce Type I errors due to clustering^96^. Also, to estimate different residual variances between the two sexes, we modelled group-wise heteroscedasticity structure, which was defined using the lme function’s argument weights = varIdent (form = ~1 | sex). We parsed a custom function to apply this model using conditional statements to account for situations where the random factor (sub-strain or metadata group) has only one unique term (e.g., data for trait ‘tibia length’ comprised only of strain C57BL/6N). In other words, some traits had only one sub-strain or one metadata group, and in such cases we did not fit the corresponding random effect. This procedure resulted in a final data set of 2,080,767 and 363 traits (i.e., the model did not converge for several traits, which were excluded from the subsequent analysis).

For each phenotypic trait, model parameters (regression coefficients and variance components) were extracted, using R package broom.mixed v.0.2.7^97^, for males and females (slope, intercept, standard error, SE of slope, SE of intercept, R^2^ marginal, R^2^ conditional and residual variance) and corresponding *p* values for regression coefficients were extracted to assess the significance of sex differences in slope and intercept. Because the lme function did not provide statistical significance for differences in residual variances (standard deviations, SDs), we used the method developed by Nakagawa et al.^98^ or the logarithm of variability ratio, which compares the difference in SDs between two groups to obtain *p* values for residual SD differences (see also Senior et al.^99^).

We were aware that some of the 363 studied traits were strongly correlated (i.e., non-independent: e.g., traits from left and right eyes and immunological assays with hierarchically clustering and overlapping cell types). Therefore, we collapsed *p* values of these related traits into 219 *p* values, using the procedure (grouping related traits or trait grouping) performed by Zajitschek et al.^18^. We employed Fisher’s method with the adjustment proposed by Li and Ji^100^ implemented in the R package, poolr^101^, which modelled the correlation between traits; we set this correlation to 0.8.

To assess the extent to which mouse sub-strain explains variation in our model, we wrote a custom function to compare our above applied model, including sub-strain as a random factor, with a model that excluded sub-strain. We extracted the difference (delta, δ) in model fit, measured using Akaike Information Criterion (AIC), and the associated *p* value for all traits that contained more than one sub-strain, included in both the applied model and the model without sub-strain (248 traits). We further applied the above approach of removing non-independent traits and collapsing *p* values (see ref. 18) for related traits, to create a reduced data set (154 traits), and conducted the same model comparisons using δAIC.

### Static allometry hypotheses and sex bias in allometric parameters

Using parameters extracted from the above models, three scenarios were assessed (see Fig. 1), describing the form of sex differences in the static allometric relationship between phenotypic trait value and body weight. For a given trait, these were: (a) males and females have significantly different slopes but share a similar intercept (Fig. 1a, d), (b) males and females have significantly different intercepts but share a similar slope (Fig. 1b, e), (c) males and females have significantly different slopes and intercepts (Fig. 1c, f). In addition, we assessed how many traits were significantly different in residuals SDs between the sexes. For these classifications, we used both *p* values from 363 traits and 219 merged trait groups.

For scenarios A–C, which represent significant differences between male and female regression slope and/or intercept parameters and cases where sex differences in SDs were significant, data were collated into functional groupings (as listed above) to assess whether, and to what extent, sex bias in parameter values and variance was present across phenotypic trait values. That is, when males and females differed significantly, we counted which sex displayed the greater parameter value (intercept, slope) and, separately, we also tallied the sex with the higher magnitude of variance. Results were pooled for phenotypic traits within a functional group and visualised using R package ggplot 2v. 3.3.5^102^ for scenarios A–C, resulting in one set of comparisons for parameter values, and one for variance (SD) values. We should highlight that we only used the data set with 363 traits because the directionality of some trait values became meaningless once traits were merged, although merged *p* values were meaningful as *p* values are not directional (e.g., spending time in light side or dark side).

### Meta-analysis of differences in slopes, intercepts and residual SD

We were aware that our classification approach using *p* values is akin to vote counting, which has limitations^103^. Therefore, we conducted formal meta-analyses using the following effect sizes: (1) difference between intercepts (traits mean for males and females on the natural log scale), (2) difference between slopes (on the log-log scale), and (3) differences between residuals SDs used corresponding SE or, more precisely, the square of SE as sampling variance (the details of this effect size can be found elsewhere^98,99^). The first two effect sizes and their sampling error variance are obtained as regression coefficients and their error variance from the mixed model described above. We note that the difference between male and female intercepts are equivalent to the log response ratio (lnRR)^104^ that shows relative difference between the two groups (note the intercept difference signifies the difference when males and females are at the average population weight). Because both types of effect sizes are on the scale pertaining to the natural logarithm, they can be compared across different traits. In addition, we calculate Cohen’s *d*, a common estimator of standardised mean difference (SMD) for each trait, along with lnRR. SMD was calculated to illustrate the magnitude of the difference in mean trait value for males and females, adopting the standard benchmarks of SMD values for small effect = 0.2, medium effect = 0.5 and large effect = 0.8 SMD (or *d*).

Our main interest in this study was whether males and females were different in intercepts, slopes and residuals SDs irrespective of effect size directionalities. Therefore, we conducted meta-analyses of magnitudes applying the transformation to the mean and sampling variance, which assumes to follow folded normal distribution (eq. 8^105^), by using the formulas below:

1 $${{{\mbox{ES}}}}_{{{{{{\rm{folded}}}}}}}={{\mbox{SE}}}\sqrt{\frac{2}{\pi }}\exp \left(-\frac{{{{\mbox{ES}}}}^{2}}{2{{{\mbox{SE}}}}^{2}}\right)+{{\mbox{ES}}}\left[1-2\varPhi \left(-\frac{{{\mbox{ES}}}}{{{\mbox{SE}}}}\right)\right]$$

2 $${{{\mbox{SE}}}}_{{{{{{\rm{folded}}}}}}}^{2}={{{\mbox{ES}}}}^{2}+{{{\mbox{SE}}}}^{2}-{{{\mbox{ES}}}}_{{{{{{\rm{folded}}}}}}}^{2}$$

where $\Phi$ is the standard normal cumulative distribution function and ES_folded_ and SE_folded_ are, respectively, transformed effect size (point estimate) and sampling variance, while ES and SE are corresponding point estimate and sampling variance before transformation. In addition to these three effect sizes, we also meta-analysed another effect size, which is the model fit quantified by marginal R^2^ (variance accounted for by fixed effects; in our case, weight, sex and their interaction). The marginal R^2^ values were squared-root transformed into correlation values, which are akin to the corelation between observed trait values and model predicted values (based on fixed effects). Then, we transformed this correlation value to Fisher’s Zr so that we could calculate sampling variance based on sample sizes (see Supplementary Material for results).

Morrissey^105^ has shown that meta-analytic means using such a folding transformation (absolute valued effect sizes) are hardly biased. Therefore, these transformed variables were directly meta-analysed using the rma.mv function in the R package, metafor^106^. The intercept models (meta-analytic model) had three random factors: (1) functional group, (2) traits group and (3) effect size identifier (which is equivalent to residuals in a meta-analytic model^107^), while in the meta-regression models, we fitted functional group as a moderator (see Fig. 3). The model structures for all the three effect sizes were identical. We reported parameter estimates and 95% confidence intervals, CI and 95% prediction intervals, PI, which were visualised as orchard plots (similar to a violin plot^108^) using the R package, orchaRd^109^. In a meta-analysis, 95% PI represents the degree of heterogeneity as well as a likely range of an effect size for a future study. We considered the estimate statistically significant when 95% CI did not span zero.

### Correlations among differences in slopes, intercepts, residual SDs and model fits

We also quantified correlations among the four effect sizes, using a Bayesian quad-variate meta-analytic model, implemented in the R package, brms^110^. We fitted functional grouping as a fixed effect and trait groups as a random effect using the function, brm. Notably, we have log transformed ES_folded_ and Zr and also transformed SE_folded_ and SE for Zr using the delta method (e.g.,^111^), accordingly, before fitting effect sizes to the model. We imposed the default priors for all the parameter estimated with the settings of two chains, 1000 warm-ups and 4000 iterations. We assessed the convergence of the chains by Gelman-Rubin statistic^112^, which was 1 for all chains (i.e., meaning they were all converged) and we also checked all effective sample sizes for posterior samples (all were over 800). We reported mean estimates (correlations among the three effect sizes and model fits) and 95% credible intervals (CI) and if the 95% CI did not overlap with 0, we considered the parameter statistically significantly different from 0. Quad-variate regressions for Zr are provided in the Supplementary Material, to illustrate the range and magnitude of the allometric fit across functional groups, and in relation to slope, intercept and SD values (Supplementary Note 1, Supplementary Fig. 1, Supplementary Fig. 2).

### Reporting summary

Further information on research design is available in the Nature Portfolio Reporting Summary linked to this article.

## Supplementary information

Supplementary Information

Description of Additional Supplementary Files

Supplementary Data 1–2

Reporting Summary

## Data Availability

The source data generated in this study have been deposited on GitHub (https://github.com/itchyshin/mice_allometry). The data used in this study are available on the Zenodo database (10.5281/zenodo.7336162)^91^. Data were compiled from International Mouse Phenotyping Consortium (IMPC) (www.mousephenotype.org, IMPC data release 10.1 June 2019).

## Code availability

An R Markdown file with instructions and the complete workflow for all analyses is provided in the supporting information, available at https://itchyshin.github.io/mice_allometry/.
