# Supplementary material for: Sex differences in allometry for phenotypic traits in mice indicate that females are not scaled males
Source: Nat Commun. 2022 Dec 12;13:7502. doi: 10.1038/s41467-022-35266-6 (PMC9744842; doi:10.1038/s41467-022-35266-6)
Supplement: Supplementary file 1 — Supplementary Information [file 41467_2022_35266_MOESM1_ESM.pdf]

Supplementary information

Sex differences in allometry for phenotypic traits in mice indicate that females are not scaled males

Laura A. B. Wilson, Susanne R. K. Zajitschek, Malgorzata Lagisz, Jeremy Mason, Hamed Haselimashhadi, Shinichi Nakagawa

Contents

Supplementary Note 1. Model Fit (Zr) Results……………………………………………………………….........................…2

Supplementary Figure 1. Orchard plots illustrating results of multivariate meta-analysis (Zr)..3

Supplementary Figure 2. Bivariate ordinations of log absolute difference between males and females for sex differences (Zr)...4

Supplementary Table 1. Point estimate (meta-analytic mean) effect sizes for orchard plots..5

Supplementary Note 1. Model Fit (Zr) Results

Effect size was highest in the morphology (0.274 [0.188-0.361, CI]) and physiology (0.228 [0.164-0.293, CI]) groups and lowest in the hearing group (0.041 [-0.111-0.194, CI]) (Supplementary Figure 1), the latter presenting a non-significant confidence interval overlapping with zero. Sex differences in variance accounted for by model fit appeared most variable for heart and morphology groups (Supplementary Figure 1b).

Results from the quad-variate meta-regressions and ordinations of the relationships between slope, intercept and model fit (Zr) (Supplementary Figure 2) indicated that both differences in intercept ($r = 0.53$) and slope between the sexes ($r = 0.37$) were significantly correlated with the model fit. As such, the greater the model fit (higher R^2 marginal value, transformed to Zr) the greater the absolute difference between male and female values for intercept and, to a lesser extent, slope. In contrast, the difference in residual variance (Supplementary Figure 2) was weakly, yet significantly, correlated ($r = 0.18$) with the model fit. Among traits with significant differences in slope between males and females (Supplementary Data 1), the highest R^2 marginal values were found for morphology traits (0.25-0.71), such as lean mass and bone mineral content, as well as traits capturing organ weight (e.g., heart weight, $R^2 = 0.54$) and several physiological and metabolic traits, that capture lipid and glucose parameters (Supplementary Data 1).

Supplementary Figure 1. Orchard plots illustrating results of multivariate meta-analysis based on differences between male and female absolute values for Zr, showing overall differences (a) and, in colour, showing separate results for each functional group (b). Orchard plots show model point estimate (black open ellipse) and associated confidence interval (CIs) (thick black horizontal line), 95% prediction intervals (PIs) (thin black horizontal line; PI represents heterogeneity), and individual effect sizes (filled circle), which are scaled by their sample size (N), the number of mice included per trait.

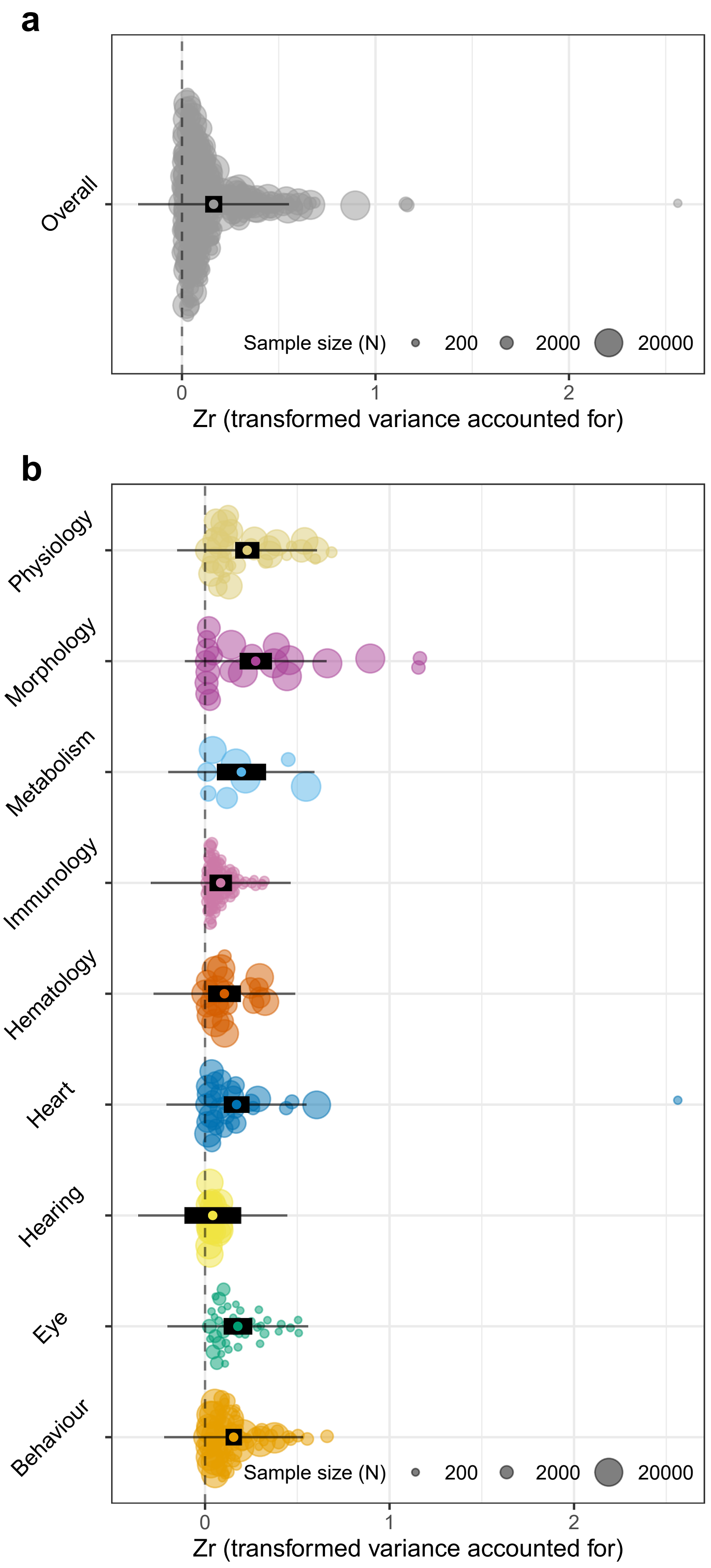

Supplementary Figure 2. Bivariate ordinations of log absolute difference between males and females for sex differences in intercept (a), slope (b) and residual SD (c) and the model fit (Zr, y-axis), for biological traits collated into nine functional groups (i.e., trait types, represented as different circle colours). Individual effect sizes (circles) are scaled by their sample size (N), the number of mice included per trait.

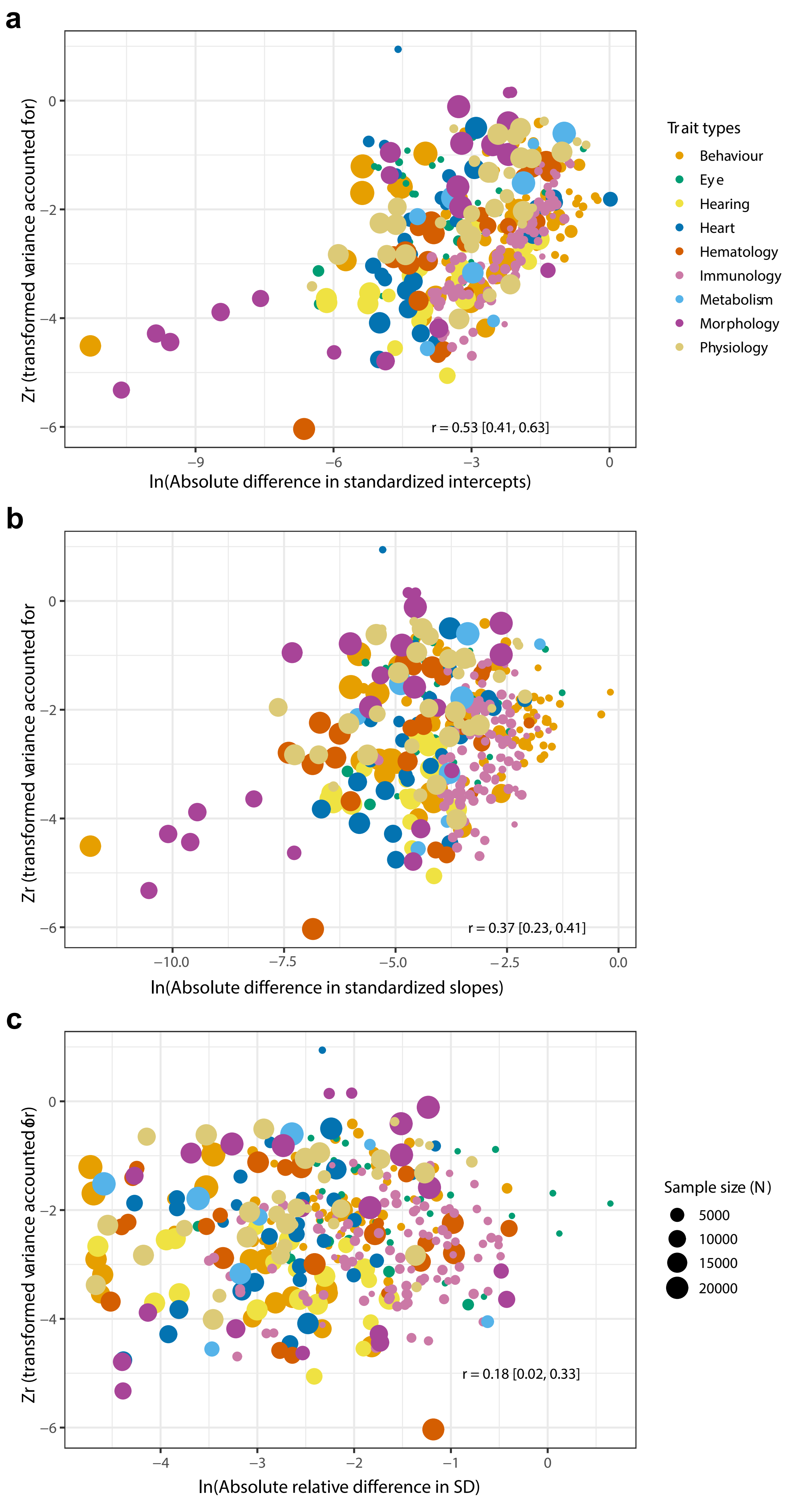

Supplementary Table 1. Point estimate effect sizes for Orchard plots (Fig. 3 main text), detailing point estimate (meta-analytic mean), confidence interval (CI), 95% prediction intervals (upper/lowerPI) and number of effect sizes (K). PI represents heterogeneity.

Figure	Comparison	Category	Effect size Point estimate	lowerCI	upperCI	lowerPI	upperPI	K
3a	Overall difference intercepts		0.089454264	0.063621	0.115287	-0.10863	0.287538	363
3b	Overall difference slopes		0.018113395	0.012578	0.023649	-0.01477	0.050998	363
3c	Overall difference SD		0.152602806	0.105427	0.199778	-0.16177	0.466973	363
3d	Difference intercepts	Behaviour	0.140508887	0.115023	0.165995	-0.04812	0.329137	85
		Eye	0.040711132	0.001518	0.079904	-0.15025	0.231675	40
		Hearing	0.049219609	-0.03393	0.132369	-0.15534	0.25378	21
		Heart	0.064155567	0.029067	0.099244	-0.12601	0.254319	31
		Hematology	0.090566763	0.045831	0.135302	-0.10161	0.282744	24
		Immunology	0.125549731	0.092557	0.158542	-0.06424	0.315338	99
		Metabolism	0.11315357	0.046144	0.180163	-0.08539	0.311701	8
		Morphology	0.046917808	0.003123	0.090712	-0.14504	0.238879	21
		Physiology	0.108545658	0.075892	0.141199	-0.08118	0.298275	34
3e	Difference slopes	Behaviour	0.021665677	0.015782	0.027549	-0.00833	0.051657	85
		Eye	0.016912874	0.008447	0.025379	-0.01369	0.047516	40
		Hearing	0.008624494	-0.00856	0.025806	-0.02544	0.042684	21
		Heart	0.011751849	0.005488	0.018016	-0.01832	0.04182	31
		Hematology	0.014143378	0.008092	0.020194	-0.01588	0.044168	24
		Immunology	0.03734789	0.028744	0.045952	0.006707	0.067989	99
		Metabolism	0.021413103	0.009496	0.03333	-0.01032	0.053144	8
		Morphology	0.009846676	0.002467	0.017227	-0.02047	0.040167	21
		Physiology	0.019321426	0.014148	0.024495	-0.01054	0.049181	34
3f	Difference SD	Behaviour	0.112091429	0.077636	0.146547	-0.17686	0.401042	85
		Eye	0.292285267	0.227599	0.356972	-0.00181	0.586377	40
		Hearing	0.08769436	-0.04036	0.215745	-0.22648	0.401864	21
		Heart	0.091460514	0.038128	0.144793	-0.20034	0.383265	31
		Hematology	0.155725329	0.086941	0.224509	-0.13929	0.450745	24
		Immunology	0.233648799	0.183984	0.283314	-0.05751	0.524805	99
		Metabolism	0.114710434	0.012588	0.216833	-0.18981	0.419234	8
		Morphology	0.160202186	0.092787	0.227617	-0.1345	0.454906	21
		Physiology	0.101224063	0.051241	0.151207	-0.18999	0.392435	34
