# Supplementary material for: Sex differences in allometry for phenotypic traits in mice indicate that females are not scaled males
Source: Nat Commun. 2022 Dec 12;13:7502. doi: 10.1038/s41467-022-35266-6 (PMC9744842; doi:10.1038/s41467-022-35266-6)
Supplement: Supplementary file 2 — Description of Additional Supplementary Files [file 41467_2022_35266_MOESM2_ESM.pdf]

Description of Additional Supplementary Files

File Name: Supplementary Data 1

Description: Comparison of variance for traits with significant slope differences between the sexes. This table is subset of all traits (n=363), filtering to include only those traits (parameter) with significant difference in slope between males and females (fm_diff_slope_p). The absolute difference in slope (fm_diff_slope), female SD (f_sd), male SD (m_sd), marginal R (r_m) are shown. The sex with the steeper slope (M=male, F=female) is noted, (i.e., fm_diff_slope = >0 females have steeper slope and where fm_diff_slope <0 males have steeper slope). The percent difference in SD is shown for comparison (% difference sd). Percent values highlighted in grey represent cases where the sex with the steeper slope shows higher variance (e.g., for parameter, left anterior chamber depth [Eye], females show a steeper slope and 32.6% of variance would be missed if fitting the male slope). All raw data are extracted from data_parameters8.csv, outputted from the supporting information R code, all workflows and data provided.

File Name: Supplementary Data 2

Description: Values of standardized mean difference (SMD), also know as Cohen's *d*, variance in standardized mean difference (v_SMD), log Response Ratio (lnRR), and variance in log Response Ratio (v_lnRR) for all 363 phenotypic traits in the dataset, compiled after initial data cleaning and filtering procedures (see Results). SMD values are shaded according to common use of effect size, showing small effect (SMD = 0.2, yellow shading), medium effect (SMD = 05, orange shading) and large effect (SMD = 0.8, blue shading). Unshaded cells show SMD of <0.2. Note that SMD are signed, such that positive effect size values indicate a greater mean for females compared to males and negative effect size values indicate greater mean for males compared to females. SMD was calculated as (female trait mean – male trait mean/ pooled SD). The exponent of lnRR (i.e., exp(lnRR)) represents percent (%) difference between males and female trait values.
