# Supplementary material for: Sex differences in allometry for phenotypic traits in mice indicate that females are not scaled males
Source: Nat Commun. 2022 Dec 12;13:7502. doi: 10.1038/s41467-022-35266-6 (PMC9744842; doi:10.1038/s41467-022-35266-6)
Supplement: Supplementary file 4 — Reporting Summary [file 41467_2022_35266_MOESM4_ESM.pdf]

nature portfolio

Corresponding author(s): Laura Wilson, Shinichi Nakagawa

Last updated by author(s): Nov 20, 2022

Reporting Summary

Nature Portfolio wishes to improve the reproducibility of the work that we publish. This form provides structure for consistency and transparency in reporting. For further information on Nature Portfolio policies, see our Editorial Policies and the Editorial Policy Checklist.

Statistics

For all statistical analyses, confirm that the following items are present in the figure legend, table legend, main text, or Methods section.

n/a	Confirmed	
☐	☒	The exact sample size (*n*) for each experimental group/condition, given as a discrete number and unit of measurement
☐	☒	A statement on whether measurements were taken from distinct samples or whether the same sample was measured repeatedly
☐	☒	The statistical test(s) used AND whether they are one- or two-sided<br>*Only common tests should be described solely by name; describe more complex techniques in the Methods section.*
☐	☒	A description of all covariates tested
☐	☒	A description of any assumptions or corrections, such as tests of normality and adjustment for multiple comparisons
☐	☒	A full description of the statistical parameters including central tendency (e.g. means) or other basic estimates (e.g. regression coefficient) AND variation (e.g. standard deviation) or associated estimates of uncertainty (e.g. confidence intervals)
☐	☒	For null hypothesis testing, the test statistic (e.g. *F*, *t*, *r*) with confidence intervals, effect sizes, degrees of freedom and *P* value noted<br>*Give P values as exact values whenever suitable.*
☐	☒	For Bayesian analysis, information on the choice of priors and Markov chain Monte Carlo settings
☐	☒	For hierarchical and complex designs, identification of the appropriate level for tests and full reporting of outcomes
☐	☒	Estimates of effect sizes (e.g. Cohen's *d*, Pearson's *r*), indicating how they were calculated

Our web collection on statistics for biologists contains articles on many of the points above.

Software and code

Policy information about availability of computer code

Data collection	No software was used to collect the data used in this study. The data were compiled from the International Mouse Phenotyping Consortium (IMPC) (www.mousephenotype.org, IMPC data release 10.1 June 2019).
Data analysis	All data procedures, along with statistical analyses, were conducted in the open source R environment v. 4.1.3. All code used in this study is freely available in the community repository, Github, linked in the Code Availability Statement. All R package versions are provided in the supplementary material, markdown file: https://itchyshin.github.io/mice_allometry/#software-and-package-versions. These are listed below.<br>attached packages: cmdstanr(v.0.5.3), rstan(v.2.21.5), StanHeaders(v.2.21.0-7), knitr(v.1.40), formatR(v.1.12), kableExtra(v.1.3.4), brms (v.2.17.0), Rcpp(v.1.0.8.3), pander(v.0.6.5), nlme(v.3.1-157), here(v.1.0.1), broom.mixed(v.0.2.9.4), orchaRd(v.2.0), patchwork(v.1.1.1), poolr (v.1.1-1), metafor(v.3.8-1), metadat(v.1.2-0), Matrix(v.1.4-1), forcats(v.0.5.2), stringr(v.1.4.1), dplyr(v.1.0.10), purrr(v.0.3.5), readr(v.2.1.3), tidyr(v.1.2.1), tibble(v.3.1.8), ggplot2(v.3.3.6) and tidyverse(v.1.3.2)<br><br>loaded via a namespace (and not attached): readxl(v.1.4.1), backports(v.1.4.1), systemfonts(v.1.0.4), plyr(v.1.8.7), igraph(v.1.3.2), splines (v.4.2.1), crosstalk(v.1.2.0), listenv(v.0.8.0), rstantools(v.2.2.0), inline(v.0.3.19), digest(v.0.6.30), htmltools(v.0.5.3), fansi(v.1.0.3), magrittr (v.2.0.3), checkmate(v.2.1.0), googlesheets4(v.1.0.1), tzdb(v.0.3.0), globals(v.0.16.1), modelr(v.0.1.9), RcppParallel(v.5.1.5), matrixStats (v.0.62.0), vroom(v.1.6.0), svglite(v.2.1.0), xts(v.0.12.1), rmdformats(v.1.0.4), prettyunits(v.1.1.1), colorspace(v.2.0-3), rvest(v.1.0.3), haven (v.2.5.1), xfun(v.0.34), callr(v.3.7.2), crayon(v.1.5.2), jsonlite(v.1.8.3), zoo(v.1.8-11), glue(v.1.6.2), gtable(v.0.3.1), gargle(v.1.2.1), emmeans (v.1.8.0), webshot(v.0.5.3), distributional(v.0.3.0), pkgbuild(v.1.3.1), abind(v.1.4-5), scales(v.1.2.1), mvtnorm(v.1.1-3), DBI(v.1.1.3), miniUI (v.0.1.1.1), viridisLite(v.0.4.1), xtable(v.1.8-4), bit(v.4.0.4), stats4(v.4.2.1), DT(v.0.23), htmlwidgets(v.1.5.4), httr(v.1.4.4), threejs(v.0.3.3), posterior(v.1.2.2), ellipsis(v.0.3.2), pkgconfig(v.2.0.3), loo(v.2.5.1), farver(v.2.1.1), sass(v.0.4.2), dbplyr(v.2.2.1), utf8(v.1.2.2), labeling(v.0.4.2), tidyselect(v.1.2.0), rlang(v.1.0.6), reshape2(v.1.4.4), later(v.1.3.0), munsell(v.0.5.0), cellranger(v.1.1.0), tools(v.4.2.1), cachem(v.1.0.6), cli (v.3.4.1), generics(v.0.1.3), broom(v.1.0.1), mathjaxr(v.1.6-0), ggridges(v.0.5.3), evaluate(v.0.17), fastmap(v.1.1.0), yaml(v.2.3.6), bit64(v.4.0.5),

processx(v.3.7.0), fs(v.1.5.2), future(v.1.28.0), mime(v.0.12), xml2(v.1.3.3), compiler(v.4.2.1), bayesplot(v.1.9.0), shinythemes(v.1.2.0), rstudioapi(v.0.14), reprex(v.2.0.2), bslib(v.0.4.0), stringi(v.1.7.8), highr(v.0.9), ps(v.1.7.1), Brobdingnag(v.1.2-7), lattice(v.0.20-45), markdown (v.1.1), shinyjs(v.2.1.0), tensorA(v.0.36.2), vctrs(v.0.4.2), pillar(v.1.8.1), lifecycle(v.1.0.3), furrr(v.0.3.0), jquerylib(v.0.1.4), bridgesampling (v.1.1-2), estimability(v.1.4.1), httpuv(v.1.6.5), R6(v.2.5.1), bookdown(v.0.26), promises(v.1.2.0.1), gridExtra(v.2.3), parallelly(v.1.32.1), codetools(v.0.2-18), colourpicker(v.1.1.1), gtools(v.3.9.2.2), assertthat(v.0.2.1), rprojroot(v.2.0.3), withr(v.2.5.0), shinystan(v.2.6.0), parallel (v.4.2.1), hms(v.1.1.2), grid(v.4.2.1), coda(v.0.19-4), rmarkdown(v.2.17), googledrive(v.2.0.0), shiny(v.1.7.1), lubridate(v.1.8.0), base64enc (v.0.1-3) and dygraphs(v.1.1.1.6)

For manuscripts utilizing custom algorithms or software that are central to the research but not yet described in published literature, software must be made available to editors and reviewers. We strongly encourage code deposition in a community repository (e.g. GitHub). See the Nature Portfolio guidelines for submitting code & software for further information.

Data

Policy information about availability of data

All manuscripts must include a data availability statement. This statement should provide the following information, where applicable:

- Accession codes, unique identifiers, or web links for publicly available datasets
- A description of any restrictions on data availability
- For clinical datasets or third party data, please ensure that the statement adheres to our policy

Source data are available on GitHub at https://github.com/itchyshin/mice_allometry, and are available in the permanent repository, Zenodo (https://zenodo.org/record/7336162). Data were compiled from the International Mouse Phenotyping Consortium (IMPC) (www.mousephenotype.org, IMPC data release 10.1 June 2019).

Human research participants

Policy information about studies involving human research participants and Sex and Gender in Research.

Reporting on sex and gender	not applicable
Population characteristics	not applicable
Recruitment	not applicable
Ethics oversight	not applicable

Note that full information on the approval of the study protocol must also be provided in the manuscript.

Field-specific reporting

Please select the one below that is the best fit for your research. If you are not sure, read the appropriate sections before making your selection.

☐ Life sciences ☐ Behavioural & social sciences ☒ Ecological, evolutionary & environmental sciences

For a reference copy of the document with all sections, see nature.com/documents/nr-reporting-summary-flat.pdf

Ecological, evolutionary & environmental sciences study design

All studies must disclose on these points even when the disclosure is negative.

Study description	We quantified the magnitude and patterning of sex differences in static allometry for adult mice phenotypic traits extracted from the IMPC database. We used a linear mixed-effects model to quantify static allometry along with a meta-analysis of differences in slopes, intercepts and residual standard deviation. Our meta-analysis used the following effect sizes: 1) difference between intercepts, quantified as trait mean for males and females, 2) difference between slopes, and 3) difference between residual SDs. Lastly, we quantified correlations among the aforementioned three effect sizes and model fit (Zr, Fishers transform from R2 marginal) using a Bayesian quad-variate meta-analytic model.
Research sample	We compiled our data set from the International Mouse Phenotyping Consortium (IMPC) (www.mousephenotype.org, IMPC data release 10.1 June 2019), accessed in October 2019. These represent traits recorded in a high-throughput phenotyping setting whereby standard operating procedures (SOPs) are implemented in a pipeline concept. The phenotypic traits represent biomarkers used for the study of disease phenotypes, collated into the following nine functional groups: behaviour, eye, hearing, heart, hematology, immunology, metabolism, morphology, and physiology, which are the IMPC's original categorization. The sample was chosen by extracting all records that contained body weight and sex information, as this research question focuses on sex differences in the relationship between phenotypic traits and body weight.
Sampling strategy	For the initial dataset, data points were collated for adult mice only, filtering to include non-categorical phenotypic trait values for which covariate information on sex and body weight were available. This initial dataset comprised of 2,866,345 data points for 419 traits, sufficient for allometric regression and the extraction of effect sizes using meta-analytical approaches. The sample represented the maximum available data extracted from IMPC data release 10.1 meeting our inclusion criteria. A series of data cleaning

	procedures were implemented to remove data points with zero values for a phenotypic trait and duplicated specimen IDs. Effective sample size was checked for posterior samples extracted from our Bayesian tri-variate meta-analytic model. These were all >800 individuals, and all chains of this model converged, evidenced by a Gelman-Rubin statistic of 1 for each convergence.
Data collection	Data were compiled for this study from the International Mouse Phenotyping Consortium (IMPC) (www.mousephenotype.org, IMPC data release 10.1 June 2019), accessed in October 2019. . Novel data collection was not undertaken. The following parameters were extracted: $external_sample_id, $phenotyping_center, $metadata_group, $biological_sample_group, $sex, $date_of_experiment, $procedure_name, $parameter_name, $data_point, $weight, $weight_days_old, $strain_accession_id, $strain_name
Timing and spatial scale	We compiled our data set from the International Mouse Phenotyping Consortium (IMPC) (www.mousephenotype.org, IMPC data release 10.1 June 2019), accessed in October 2019. This time frame represented the initiation of the research project.
Data exclusions	A series of data cleaning procedures were implemented to remove data points with zero values for a phenotypic trait and duplicated specimen IDs. These criteria were used because this research uses a linear mixed effects model for static allometry, which requires trait and body weight data to be fit, therefore specimens missing either of these variables cannot be included in the model.
Reproducibility	Experiments were not conducted in this study. All code is provided to reproduce all results presented in this study. All R package versions are listed.
Randomization	For each phenotypic trait, we had the following variables (covariates): phenotyping center name (location where experimental data were collected), external sample ID (animal ID), metadata group (identifier for experimental conditions in place during the experiment), sex (male / female), weight (body weight in grams), weight days old (day on which weight was recorded), procedure name (description of the experimental procedure as in IMPReSS), parameter name (description of the recorded parameter as in IMPReSS), and data point (phenotypic trait measurement – response variable).
Blinding	Blinding was not undertaken as it is not relevant to this study, which comprised meta-analytic procedures undertaken on 2.1million measurements. Data were compiled from release 10.1 June 2019 using the following inclusion parameters (needed for the static allometric regressions): $external_sample_id, $phenotyping_center, $metadata_group, $biological_sample_group, $sex, $date_of_experiment, $procedure_name, $parameter_name, $data_point, $weight, $weight_days_old, $strain_accession_id, $strain_name

Did the study involve field work? ☐ Yes ☒ No

Reporting for specific materials, systems and methods

We require information from authors about some types of materials, experimental systems and methods used in many studies. Here, indicate whether each material, system or method listed is relevant to your study. If you are not sure if a list item applies to your research, read the appropriate section before selecting a response.

Materials & experimental systems

n/a	Involved in the study
☒	☐ Antibodies
☒	☐ Eukaryotic cell lines
☒	☐ Palaeontology and archaeology
☒	☐ Animals and other organisms
☒	☐ Clinical data
☒	☐ Dual use research of concern

Methods

n/a	Involved in the study
☒	☐ ChIP-seq
☒	☐ Flow cytometry
☒	☐ MRI-based neuroimaging
